# Targeted C•G-to-T•A base editing with TALE-cytosine deaminases in plants

**DOI:** 10.1186/s12915-024-01895-0

**Published:** 2024-04-29

**Authors:** Dingbo Zhang, Vanessa Pries, Jens Boch

**Affiliations:** Leibniz University Hannover, Institute of Plant Genetics, Herrenhäuser Str. 2, Hannover, 30419 Germany

**Keywords:** DddA, Genome editing, Deaminase, Base editors, Rice, Chloroplast

## Abstract

**Background:**

TALE-derived DddA-based cytosine base editors (TALE-DdCBEs) can perform efficient base editing of mitochondria and chloroplast genomes. They use transcription activator-like effector (TALE) arrays as programmable DNA-binding domains and a split version of the double-strand DNA cytidine deaminase (DddA) to catalyze C•G-to-T•A editing. This technology has not been optimized for use in plant cells.

**Results:**

To systematically investigate TALE-DdCBE architectures and editing rules, we established a β-glucuronidase reporter for transient assays in *Nicotiana benthamiana*. We show that TALE-DdCBEs function with distinct spacer lengths between the DNA-binding sites of their two TALE parts. Compared to canonical DddA, TALE-DdCBEs containing evolved DddA variants (DddA6 or DddA11) showed a significant improvement in editing efficiency in *Nicotiana benthamiana* and rice. Moreover, TALE-DdCBEs containing DddA11 have broader sequence compatibility for non-TC target editing. We have successfully regenerated rice with C•G-to-T•A conversions in their chloroplast genome, as well as *N. benthamiana* with C•G-to-T•A editing in the nuclear genome using TALE-DdCBE. We also found that the spontaneous assembly of split DddA halves can cause undesired editing by TALE-DdCBEs in plants.

**Conclusions:**

Altogether, our results refined the targeting scope of TALE-DdCBEs and successfully applied them to target the chloroplast and nuclear genomes. Our study expands the base editing toolbox in plants and further defines parameters to optimize TALE-DdCBEs for high-fidelity crop improvement.

**Supplementary Information:**

The online version contains supplementary material available at 10.1186/s12915-024-01895-0.

## Background

Genome-editing technologies are rapidly revolutionizing plant breeding. Base editing is a significant innovation in the genome editing field. Instead of generating double-stranded DNA (dsDNA) breaks, base editors utilize DNA deaminases to precisely incorporate single nucleotide variants (SNVs) into the genome [1]. Current base editors generally contain a CRISPR-Cas nickase linked to a single-stranded DNA (ssDNA) deaminase enzyme. Cytosine base editors (CBEs) catalyze the transition mutation C•G-to-T•A through the deamination of deoxycytidine to deoxyuridine. The U•G mismatch can be repaired to U•A resulting in a T•A base pair. Conjugating the uracil glycosylase inhibitor (UGI) to CBEs increases the editing efficiency and purity [2–4]. In addition, C•G-to-G•C base editors have been developed by replacing the UGI with uracil N-glycosylase in the CBE architecture [5, 6]. Adenine base editors (ABEs) catalyze the transition mutation of A•T-to-G•C through the deamination of deoxyadenosine to deoxyinosine, which is analogous to guanine (G) in base pairing [7].

Although the CRISPR/Cas-derived base editors have proven to provide an efficient and precise introduction of SNVs in the nuclear genome, repurposing them for organellar (mitochondria and plastids) DNA editing is challenging due to the lack of methods for delivering sgRNAs to the mitochondria and plastids. However, protein-only genome editing systems based on DNA-binding proteins such as zinc fingers [8] and transcription activator-like effectors (TALE) [9] can be used as organellar genome editors when fused to the dsDNA-specific cytosine deaminase DddA from *Burkholderia cenocepacia* [10, 11]. To generate TALE-derived DddA-based cytosine base editors (TALE-DdCBEs), DddA was split into two inactive halves, DddA-N and DddA-C. The two DddA halves are fused to two TALE arrays which bind to targeted sequences in a tail-to-tail orientation to reconstitute the active DddA enzyme [12–15]. Subcellular targeting signals (nuclear localization signal or mitochondrial targeting signal) are fused to the N-terminus and UGI is fused to the C terminus of the TALE-DdCBEs to increase the editing efficiency and inhibit uracil-DNA glycosylase. Instead of using the split DddA halves, non-toxic, full-length DddA variants were developed to make monomeric TALE-DdCBEs (DddA guided by one TALE protein) which also allow C•G-to-T•A editing in mitochondrial DNA [16]. To improve the deamination activity and address the rigid 5′-TC context limitation of DddA, evolved DddA variants (DddA6 and DddA11) with improved activity and expanded targeting scope were created by protein engineering [17]. In plants, TALE-DdCBEs were successfully used for editing the plastid genome of *Arabidopsis* [18] and the chloroplast and mitochondrial DNA of lettuce, rapeseed [19], and rice chloroplasts [20]. Recently, Nakazato et al. reported that TALE-DdCBEs, which contain a modified version of DddA11, exhibit a high frequency of C•G-to-T•A editing in the *Arabidopsis* plastid genome [21]. Besides CBEs, TALE-based ABEs (TALEDs) have also been developed to perform mitochondrial A•T-to-G•C base editing in mammalian mitochondria [22] and *Arabidopsis* chloroplast genes [23].

In this study, we developed a modular cloning (MoClo) pipeline for TALE-DdCBEs assembly and established a simple β-glucuronidase (GUS) reporter assay in *N. benthamiana* for C•G-to-T•A editing efficiency evaluation. Using this, we characterized the size of the spacer region between TALE-DdCBE binding sites and the optimal position of the target cytosine. To validate our TALE-DdCBEs in plants, we targeted the rice chloroplast gene *OspsaA* and the *N. benthamiana* nuclear gene *NbSuRB*, and successfully generated chloroplast-edited rice and nuclear-edited *Nicotiana benthamiana* plants, respectively.

## Results

### Architecture to optimize TALE-DdCBEs editing activity

**Fig. 1:**
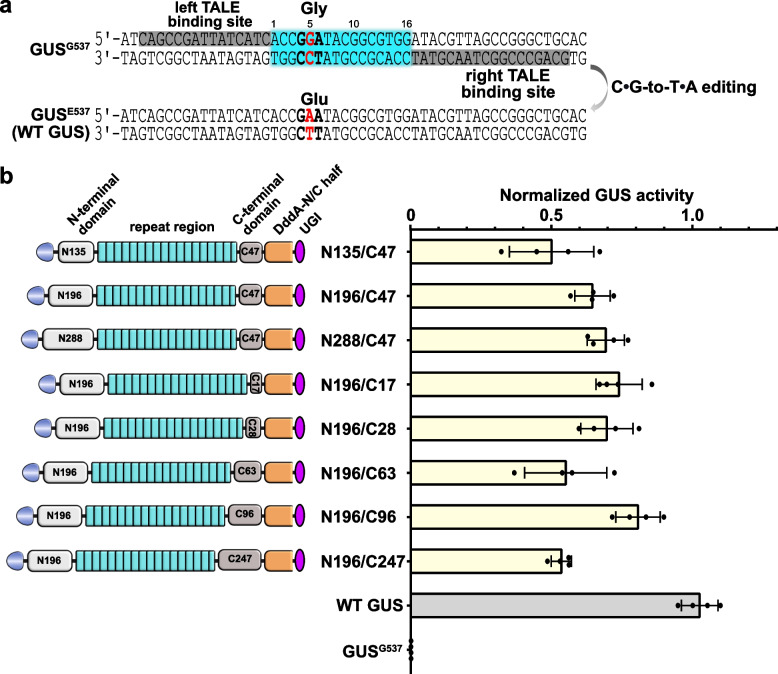
Establishment of TALE-DdCBEs in *N. benthamiana*. **a** Schematic of the GUS^G537^ cytosine base editing reporter. The C•G-to-T•A (highlighted in red) editing in GUS^G537^ can alter the glycine codon (GGA) to a glutamic acid codon (GAA) and restore GUS activity. TALE-binding sites are in gray background, and the spacer region is in cyan background. **b** Left panel: schematic of TALE-DdCBEs containing different lengths of N- and C-terminal domains. N288, 288 amino acids full-length N-terminus; N196, 196 amino acids length truncated from the N-terminal end; N135, 135 amino acids length of truncated N-terminal end. C-terminal domains truncated from the C-terminal end: C247 has 247 amino acids, C96 has 96 amino acids, C63 has 63 amino acids, C47 has 47 amino acids, C28 has 28 amino acids, and C17 has 17 amino acids. bpNLS, bipartite nuclear localization sequence; UGI, uracil glycosylase inhibitor. Right panel: C•G-to-T•A editing efficiencies of different TALE-DdCBE architectures (left TALE-DddA-C/right TALE-DddA-N) in GUS.^G537^. GUS activities were measured and normalized to 2 × 35S::GUS (WT GUS, positive control). Values and error bars indicate the mean ± SEM, *n* = 4

We first developed a GUS reporter system in *N. benthamiana* to quickly assess the C•G-to-T•A conversion efficiencies of various TALE-DdCBE architectural designs. For this, we constructed an inactive GUS^G537^ allele with a missense mutation of glutamic acid (GAA) to glycine (GGA) in the GUS enzyme active center [24]. A C•G-to-T•A conversion reverted the glycine residue to glutamic acid and restored GUS enzymatic activity (Fig. 1a and Additional file 1: Fig. S1). This demonstrates that our reporter system is suitable for analyzing TALE-DdCBE activity.

Zhang et al. reported that the length of the TALE N-terminus or C-terminus could affect TALE activity [25]. Hence, we employed three different lengths of N-terminal and six different lengths of C-terminal domains to develop a series of TALE-DdCBEs (Fig. 1b). The activities of these TALE-DdCBEs were examined using the GUS^G537^ reporter through *Agrobacterium*-mediated transient expression in *N. benthamiana* leaves. Three different N-terminal architectures (N288, N196, and N135) showed comparable GUS activity while coupled with the same C-terminal domain (C47) (Fig. 1b). N196 (196 amino acids in length with a deletion of 93 amino acids from the full-length N-terminus) was chosen for all subsequent studies. Similar analyses using truncations from the C terminus demonstrated that N196/C17 (C17: C-terminal domain of 17 amino acids in length) and N196/C96 (C96: C-terminal domain of 96 amino acids in length) have the highest GUS activity, whereas N196/C247 (C247: 247 amino acids in length resembling the full-length C terminus without the native transcriptional activation domain) showed the lowest GUS activity. To minimize the size of TALE-DdCBEs and maintain its high editing efficiency, we selected N196/C17 as the optimal TALE N-terminal and C-terminal combinations and used these for the following experiments.

### Characterization of the TALE-DdCBE editing window

**Fig. 2:**
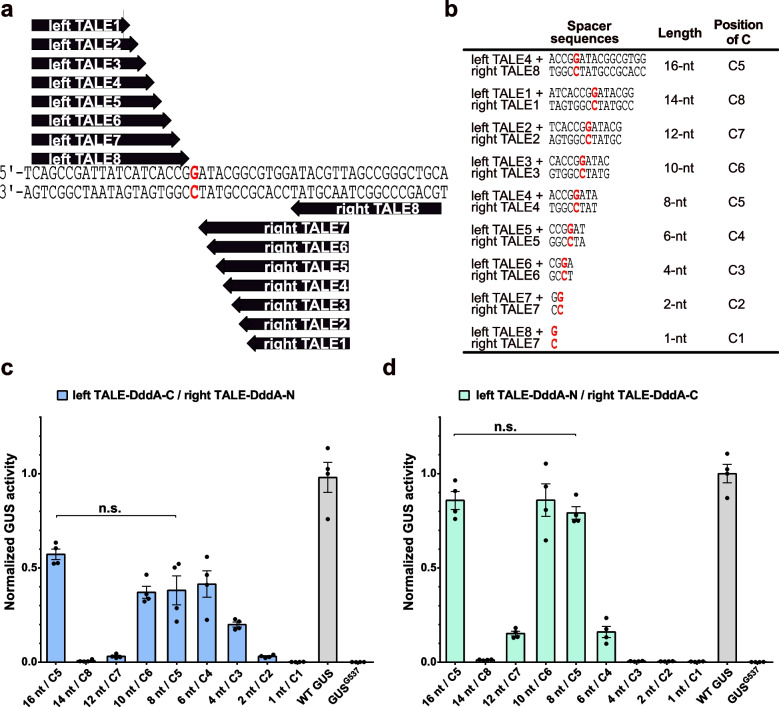
Editing windows of a pair of TALE-DdCBEs. **a** Schematic of shifting the spacer region between a pair of TALE-DdCBEs and the position of the target cytosine by using TALE arrays of different lengths. The binding sites of eight left TALEs and eight right TALEs are shown by arrows. The targeted C•G base pair is in red. **b** Different spacer regions (from 1 to 16 nt) flanked by different left and right TALE combinations. The targeted cytosine is in red and bold. **c**, **d** C•G-to-T•A editing efficiencies of TALE-DdCBEs using left TALE-DddA-C/right TALE-DddA-N architectures (**c**) or left TALE-DddA-N/right TALE-DddA-C architectures (**d**) in the GUS^G537^ reporter. GUS activities were measured and normalized to 2 × 35S::GUS (WT GUS, positive control). Values and error bars indicate the mean ± SEM, *n* = 4, n.s. (not significant) using Student’s two-tailed unpaired *t*-test

To further investigate how a pair of TALE-DdCBEs should be positioned to modify a specific target base, we constructed TALE arrays of different lengths flanking the targeted cytosine in the GUS^G537^ reporter (Fig. 2a). The different combinations of left and right TALE DNA-binding domains enable the evaluation of spacer regions ranging from 1 to 16 nt in length. The position of the targeted cytosine within the spacer was varied from position 1 to position 8 (C1 to C8) (Fig. 2b). The DddA-C half was fused to the left TALE (left TALE-DddA-C) coupled with the DddA-N half fused to the right TALE (right TALE-DddA-N) or the opposite way (left TALE-DddA-N and right TALE-DddA-C). We tested the editing efficiencies of those TALE-DdCBEs in *N. benthamiana* using the GUS^G537^ reporter. In the left TALE-DddA-C/right TALE-DddA-N architecture, the highest efficiencies were achieved at cytosines positioned at C4, C5, or C6 across different spacer sizes (Fig. 2c). For the left TALE-DddA-N/right TALE-DddA-C architecture, the highest editing efficiency was achieved at the targeted cytosine located at C5 or C6 (Fig. 2d). When the targeted cytosines were at positions C7 or C8, the editing efficiencies were dramatically decreased using either left TALE-DddA-C/right TALE-DddA-N or left TALE-DddA-N/right TALE-DddA-C architectures. These results indicated that TALE-DdCBEs prefer the target cytosine to be located at C5 or C6.

**Fig. 3:**
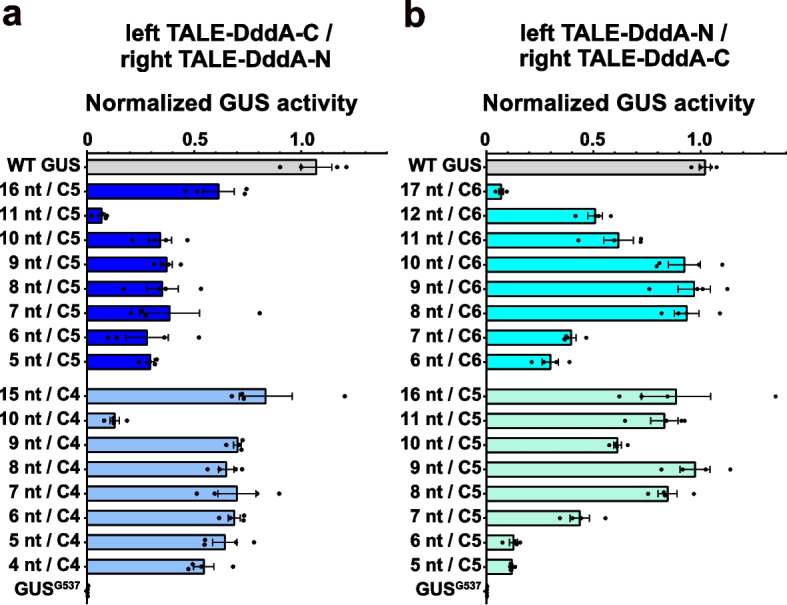
Editing efficiencies of TALE-DdCBEs with different spacer lengths. **a** C•G-to-T•A editing efficiencies of the targeted cytosine at C4 or C5 in spacers of different lengths using left TALE-DddA-C/right TALE-DddA-N architectures in the GUS^G537^ reporter. **b** C•G-to-T•A editing efficiencies of the targeted cytosine at C5 or C6 in spacers of different lengths using left TALE-DddA-N/right TALE-DddA-C architectures in the GUS^G537^ reporter. GUS activities were measured and normalized to 2 × 35S::GUS (WT GUS, positive control). Values and error bars indicate the mean ± SEM, *n* = 4

To further characterize the optimal spacer length for TALE-DdCBEs, we analyzed their editing efficiencies when targeting the cytosines located at C4, C5, or C6 in different sizes of spacing regions. In both orientations, the spacer lengths could be dramatically reduced, while containing full or at least significant activity (Fig. 3a, b). Even an extremely short spacer of only 4 nt in length allowed substantial editing of the C4 position (Fig. 3a). Taken together, these findings reveal that when the TALE-DddA-C half is binding to the DNA strand harboring the target cytosine and the TALE-DddA-N half binding to the other strand, the optimal editing efficiency is at C5 or C6 in a 9-nt spacer region.

### DddA variants show high activities in plant cells

It has been reported that fusing a single-strand DNA-binding domain from RADIATION SENSITIVE 51 (Rad51) to a cytosine base editor [26] or adenine base editor [27, 28] could enhance the base editing capability in mammalian cells and rice. We wondered if the fusion of Rad51 could influence the editing efficiency of TALE-DdCBEs; therefore, we inserted the Rad51 domain before the DddA halves or before the TALE N-terminal domain (Additional file 1: Fig. S2). GUS reporter assays showed that by using the N196/C17 or N196/C96 TALE architectures, the fusion of Rad51 did not increase but, in some combinations, even decreased the editing efficiency of TALE-DdCBEs.

Recently, Mok et al. used phage-assisted continuous evolution (PACE) to evolve the DddA protein and isolated the variants DddA6 (Q1310R, S1330I, T1380I, T1413I) and DddA11 (S1330I, A1341V, N1342S, E1370K, T1380I, T1413I) with fourfold higher C•G-to-T•A editing efficiencies [17]. Moreover, DddA11 enabled editing of CC, AC, and GC targets whereas the wild-type DddA has a strict TC target preference. Hence, we replaced the DddA-C/N halves with DddA6-C/N halves or DddA11-C/N halves in our TALE base editors and tested them using the GUS^G537^ reporter. Furthermore, we combined the point mutations of DddA6 and DddA11 and generated a new DddA variant named DddA611. GUS reporter assays showed that the editing activities of DddA6, DddA11, and DddA611 are dramatically increased in comparison with WT DddA (Additional file 1: Fig. S3). We would like to point out that the GUS assay is only an approximation for the TALE-CBE activity. The GUS^G537^ reporter contains several cytosines in the spacer region of which only one is the target for the glycine to glutamic acid exchange that restores GUS activity. While this is the only one in a TC context that is strongly preferred by the wild-type DddA, we can not exclude that alternative C-to-T transitions might compromise GUS activity.

**Fig. 4:**
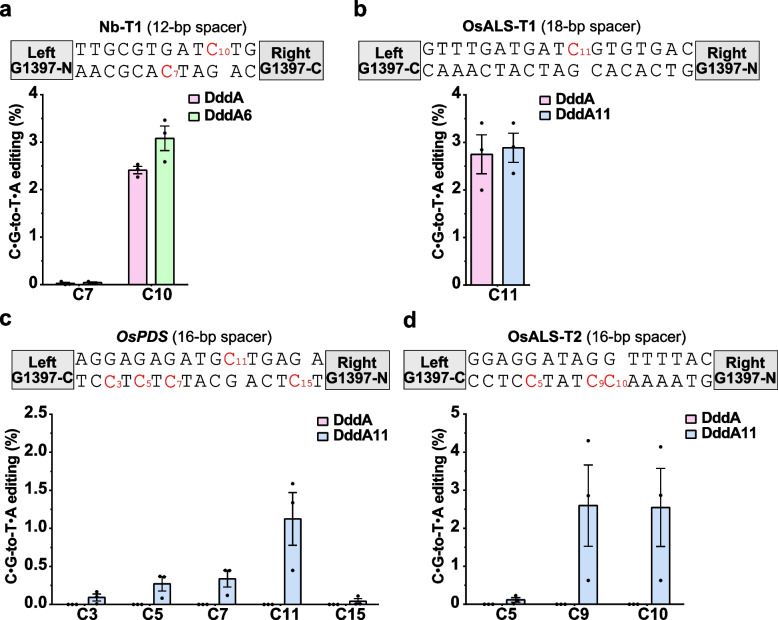
Editing efficiencies of TALE-DdCBEs in rice and *N. benthamiana* protoplasts. **a**–**d** C•G-to-T•A editing efficiencies of TALE-DdCBEs were determined by amplicon sequencing of target regions from *N. benthamiana* (**a**) and rice protoplasts (**b**–**d**). Targeted sequences are listed above the panels. Values and error bars indicate the mean ± SEM, *n* = 3 independent experiments

To further characterize the targeting capabilities of DddA, DddA6, and DddA11, we targeted the tobacco gene (Nb-T1) and the rice genes (OsALS-T1, OsALS-T2, and *OsPDS*) in protoplasts. Amplicon sequencing showed that DddA achieved C•G-to-T•A editing efficiency of approximately 2.4% of C10, while DddA6 yielded an editing efficiency of around 3.1% at the Nb-T1 locus (Fig. 4a). At the OsALS-T1 target site, both DddA and DddA11 showed similar editing efficiency at C11 in a TC context (Fig. 4b). For *OsPDS* and OsALS-T2, DddA showed no editing efficiency within the spacer regions, whereas DddA11 showed high editing efficiencies of multiple cytosines in *OsPDS* (C3, C5, C7, C11) (Fig. 4c), as well as in OsALS-T2 (C9, C10) (Fig. 4d). Notably, DddA11 exhibited non-TC target editing activities of GC (C11; 1.1%), CC (C9; 2.6%), and AC (C10; 2.5%) at these two target sites. These results show that the three DddA variants DddA6, DddA11, and DddA611 substantially increase the C•G-to-T•A editing efficiency in plant cells and DddA11 enables editing of non-TC targets.

### Spontaneous assembly of the split DddA halves

**Fig. 5:**
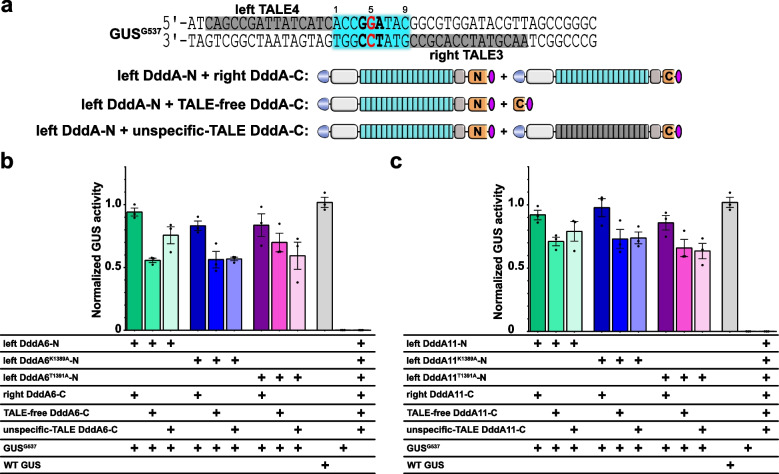
Off-target editing of TALE-DdCBEs using the GUS^G537^ reporter. **a** TALE-DdCBE constructs were used to target the GUS^G537^ reporter. TALE-binding sites are in gray background, and the spacer region is in cyan background. The targeted cytosine is located at C5 within the 9-nt spacer. **b** C•G-to-T•A editing efficiencies of TALE-DdCBEs containing DddA6 or its variants DddA6^K1389A^ or DddA6^T1391A^. **c** C•G-to-T•A editing efficiencies of TALE-DdCBEs containing DddA11 or its variants DddA11^K1389A^ or DddA11.^T1391A^. GUS activities were measured and normalized to 2 × 35S::GUS (WT GUS, positive control). Values and error bars indicate the mean ± SEM, *n* = 3

TALE-DdCBE-mediated base editing in the mitochondria can generate off-target editing, both in the mitochondria and nuclear chromosomes [29, 30]. It has been reported that spontaneous assembly of DddA halves can cause undesired editing in mammalian cells [31]. Here, we profiled possible spontaneous assembly of DddA halves in plant cells using the GUS^G537^ reporter. Two situations were analyzed. First, only one TALE array of the TALE-DdCBE pair binding to a given site (the GUS^G537^ reporter) while the other is not (unspecific TALE domain), and, second, one TALE array of the TALE-DdCBE binding to the GUS^G537^ reporter with the second half of DddA provided as an independent domain (TALE-free) (Fig. 5a). GUS activities in *N. benthamiana* showed that the two off-target scenarios show 55.7% (left DddA6-N/TALE-free DddA6-C) and 75.6% (left DddA6-N/unspecific TALE DddA6-C) activities, compared to 94% activity of the on-target situation (left DddA6-N/right DddA6-C) (Fig. 5b). For DddA11, the off-target scenarios showed editing efficiencies of 71% and 79%, compared to 92% on-target activity (Fig. 5c). These data indicate that the spontaneous assembly of split DddA halves is sufficient to trigger C•G-to-T•A editing at loci where one TALE array is binding.

To avoid spontaneous assembly of split DddA halves, a high-fidelity TALE-DdCBE was recently developed by substituting amino acid residues at the interface between the split DddA halves with alanine (K1389A or T1391A) [31]. We wondered whether these high-fidelity TALE-DdCBEs could prohibit the assembly of functional DddA in the absence of properly placed TALE-DNA interaction in plants. For this, the K1389A or T1391A mutation was introduced into DddA6 (DddA6^K1389A^/DddA6^T1391A^) and DddA11 (DddA11^K1389A^/DddA11^T1391A^). The specificities of these high-fidelity DddA variants were analyzed using the *N. benthamiana* GUS^G537^ reporter. Surprisingly, DddA6^K1389A^, DddA6^T1391A^, DddA11^K1389A^, and DddA1^T1391A^ showed similar editing efficiencies compared to DddA6 using the two off-target scenarios (Fig. 5b, c). Together, these results indicate that unlike those in the mitochondria [31], the high-fidelity DddA mutants could not reduce the spontaneous assembly of split DddA halves as well as off-target editing in our reporter assay in plant cells.

### TALE-DdCBE-mediated C•G-to-T•A conversion in plants

**Fig. 6:**
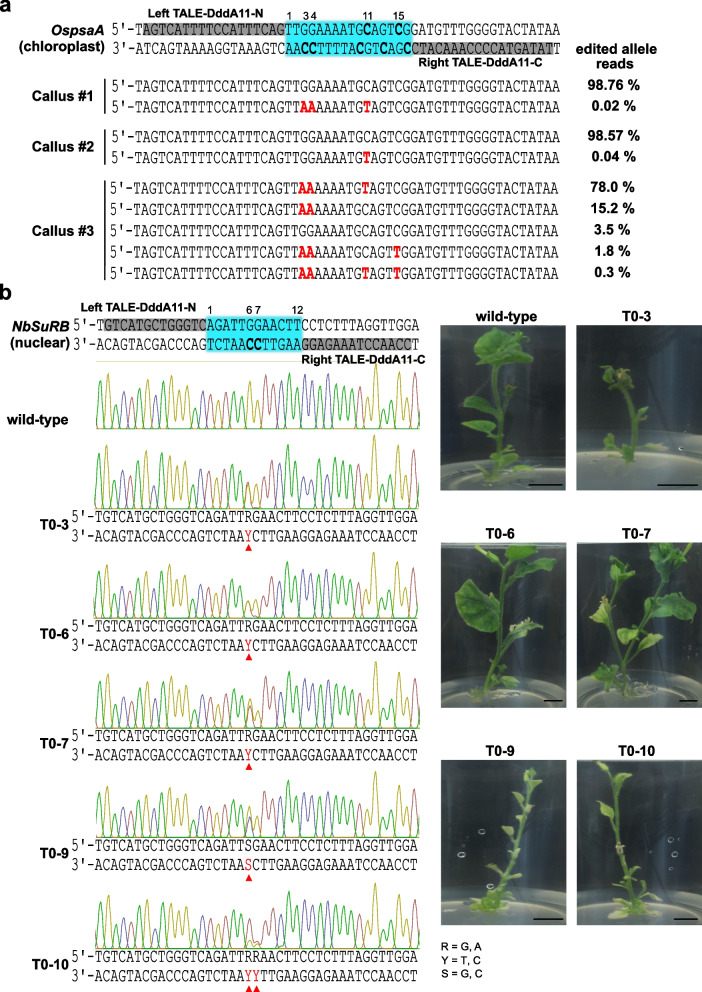
Organellar and nuclear DNA editing by TALE-DdCBE. TALE-DdCBEs targeting the chloroplast genome in rice and the nuclear genome in *N. benthamiana*. **a** A pair of TALE-DddA11 targeting the rice *OspsaA* chloroplast gene. TALE-binding sites are in gray background, and the spacer region is in cyan background. Amplicon deep sequencing results from regenerated calli. Frequencies and edited patterns induced by TALE-DddA11 are shown. **b** A pair of TALE-DddA11 targeting the *NbSuRB* nuclear gene in tobacco. TALE-binding sites are in gray background, and the spacer region is in cyan background. Sanger sequencing chromatograms of wild-type and regenerated T0 plants are shown. Base conversions are indicated in red and marked by red triangles. Bar = 1 cm

To demonstrate the efficiency of the TALE-DddA11 fusion in converting C•G-to-T•A in rice organelles, we targeted the chloroplast gene *OspsaA*. We analyzed three independent regenerated rice calli and found one that contained three C•G pairs that were substituted to T•A pairs in a 16-nt spacer region (Fig. 6a). Amplicon deep sequencing revealed chimeric C•G-to-T•A conversions in callus #3 with C3, C4, C11, and C15 edited. Specifically, C3, C4, C11, and C15 were found in a CC, TC, GC, and TC sequence context, respectively, while the other two calli showed nearly no editing. Moreover, we found that one regenerated plant (T0-psa-1) showed a light green coloring of the leaves and stem compared to the wild-type plant. Sanger sequencing results indicated nearly homoplasmic C•G-to-T•A editing of C3 and C4, which generated a premature stop codon (TAA) from the tryptophan codon (TGG) (Additional file 1: Fig. S4).

These results show that TALE-DdCBEs can efficiently target chloroplast genes.

Nuclear genome editing using TALE-DdCBEs in plants has not been reported before. To investigate the nuclear genome editing ability of TALE-DdCBEs, we designed a pair of TALE-DddA11 fusion constructs targeting the *N. benthamiana* acetolactate synthase gene *NbSuRB*. Sanger sequencing results showed that five out of ten regenerated plant lines contained an edited C•G pair in the 12-nt spacer region (Fig. 6b). Among the five edited lines, T0–3, T0–6, and T0–7 harbored heterozygous C•G-to-T•A conversions at C6 (CC context), and T0–10 contained heterozygous C•G-to-T•A conversions at both C6 (CC context) and C7 (TC context). Instead of a C•G-to-T•A conversion, line T0–9 contained a heterozygous C•G-to-G•C mutation at C6. This byproduct might have been caused by base excision repair. To determine whether TALE-DddA11 can induce heritable C•G-to-T•A conversion events, we randomly selected two or four individual T1 seedlings from four different T0 lines for genotyping. Sanger sequencing results showed stable inheritance of C•G-to-T•A (T0–6, T0–7, and T0–10) or C•G-to-G•C (T0–9) conversions in these T1 lines. Moreover, homoallelic mutations caused by segregation were detected in the T1 populations (Additional file 1: Table S1). To analyze potential off-target editing, we sequenced the top 5 predicted TALE-dependent off-target sites in three different T0 lines (T0–3, T0–6, and T0–10). No off-target editing was detected in these plants (Additional file 1: Table S2). Together, these results demonstrate that DddA11 enables editing of non-TC targets, and TALE-DddA11 can generate C•G-to-T•A editing in both the chloroplast and the nuclear genome of plants.

## Discussion

In this study, we further characterized TALE-DdCBE architectures and applied them in plants. We determined the optimal length of the spacer as well as the preferential position of the target cytosine. These results have the potential to improve the precision and efficiency of TALE-DdCBEs in plants. To streamline these experiments, we developed a MoClo system to allow a variable assembly of TALE-DdCBEs using Golden Gate Cloning [32] and established a GUS reporter as a simple way to quantify their C•G-to-T•A editing efficiencies in plant cells.

Using this, we tested different architectures of N- and C-terminal TALE domains. The N-terminal 152 amino acids can be deleted from TALEs without abolishing their function inside eukaryotic cells [33], and many biotechnological uses of TALEs, e.g., as TALEN, apply this truncated N-terminal domain (N135). Deleting larger regions interferes with the non-specific DNA-binding region in the N-terminal domain [34]. On the other hand, a slightly larger region (N196) was shown to confer enhanced DNA-binding activity without residual transcriptional activation activity [35, 36]. In contrast, the C-terminal domain of TALEs does not contribute to DNA binding and can be truncated to short fragments (C17) [37]. Based on our GUS reporter results, we recommend the use of N196 and C17 as N- and C-terminal combinations, respectively, in TALE-DdCBE architectures to minimize protein size without sacrificing activity.

Defining the appropriate editing window is crucial when employing TALE-DdCBEs, which corresponds to the space length between the two TALE-binding sites and the target cytosine position within this spacer. In mammalian cells, the TALE-DdCBEs (G1397-split DddA) preferentially edited TCs that were located approximately 4–7 (C4-C7) nucleotides upstream of the 3′ end of spacer regions ranging from 14 to 18 nt in mitochondrial DNA [10] or 11 to 17 nt in nuclear targets [38]. TALE-DdCBEs containing either WT DddA or DddA6/DddA11 showed similar editing windows [17]. In our GUS reporter experiments, we found that the orientations of G1397-split DddA halves (the DddA-N and DddA-C halves fused either to the left or right TALE, respectively) slightly affected the editing window. The window ranged from C3 to C6 or C4 to C7, dependent on the orientations. In conclusion, we suggest targeting the C5 and C6 positions upstream of the 3′ end of the spacer region as the preferred choice.

Furthermore, we detected significant base editing activity of TALE-DdCBEs at spacer regions which are significantly shorter than the originally anticipated 14 to 18 nt [10]. The GUS assay detected editing activity down to a minimal spacer of 4 nt, but for optimal activity, a spacer no shorter than 8 nt should be used. A 9-nt spacer for target cytosines at position C5 or C6 appears to be optimal.

TALE-DdCBEs containing WT DddA have a strong preference for editing TC contexts [10] which limits possible targets of TALE-DdCBEs in plant genome editing. Consistent with previous studies in mammalian cells [17], we show that the utilization of DddA variants (DddA6, DddA11) enhances the TALE-DdCBEs editing activity at TC targets also in plant cells. Furthermore, DddA11 enabled efficient editing of non-TC targets in plant cells which significantly expands the targeting scope of TALE-DdCBEs. As a less restricted alternative, it was recently reported that a DddA homolog from *Simiaoa sunii* (Ddd_Ss) can efficiently deaminate cytosine at non-TC targets [39]. It will be helpful to compare the editing capabilities of DddA11 and Ddd_Ss in future studies of TALE-DdCBEs in plants.

TALE-DdCBEs directed to the mitochondria have been reported to result in off-target editing in the mitochondria and the nucleus [29, 30]. The occurrence of off-target editing on nuclear DNA suggests that a mitochondrial targeting signal (MTS) is ineffective in preventing the entry of TALE-DdCBEs into the nucleus of mammalian cells. It has been proposed that the addition of a nuclear export signal (NES) to TALE-DdCBEs could mitigate off-target editing in the nuclear genome [15, 30]. Off-target editing of TALE-DdCBEs can be attributable to two main factors: non-specific TALE-DNA interactions and spontaneous reassembly of the split DddA halves. The former is dependent on TALE proteins and target sequence selection, as some TALE binding sites may share similar sequences in the genome. Meanwhile, the latter represents a more pervasive issue. We analyzed five predicted TALE-dependent off-target sites in three independent T0 plants and did not detect any off-target editing. Nevertheless, in agreement with previous studies in mammalian cells [30, 31], we show that the undesired editing can be caused by spontaneous assembly of DddA halves guided by one TALE array in our GUS^G537^ reporter studies in plant cells. DddA and its variants DddA6 and DddA11 containing K1389A or T1391A point mutations have been reported to restrict the spontaneous assembly of DddA halves and limit off-target editing in human mitochondrial DNA [31]. However, we found that these variants did not show a significant decrease in unspecific editing using our GUS^G537^ reporter. This conflicting result might be due to experimental differences between the mitochondrial DNA and our plant transient reporter system. It is possible that the GUS reporter is more sensitive and might, therefore, overestimate the actual editing events.

In this study, we demonstrate that TALE-DdCBE containing the DddA11 variant can efficiently target both the chloroplast genome and the nuclear genome in plants. Our studies show for the first time that it is possible to obtain full plants carrying target edited sites. Hence, the TALE-DdCBE system can be considered as an alternative to the CRISPR/Cas-CBE system for inducing C•G-to-T•A conversions in the nuclear genome of plants. Nevertheless, we identified only heterozygously edited T0 plants when targeting the nuclear genome. TALE-DdCBEs, in contrast to nickase-Cas9 cytosine base editors, are unable to induce a nick in the non-deaminated DNA strand. Accordingly, TALE-CBEs do not trigger the activation of cellular repair mechanisms that selectively replace the nicked strand and utilize the deaminated strand as a template for repair. Recently, researchers tethered a nickase to a TALE-deaminase. This TALE-nickase base editor was shown to be highly active in mammalian cells [40] and rice protoplasts [41]. Future studies will show if an additional nickase can also enhance the editing rate of genomic sites in regenerated plants. The use of CRISPR/Cas-based base editors has been successful in accurately and effectively introducing single nucleotide variants (SNVs) into the nuclear genome. However, adapting this technology for editing DNA in organelles such as mitochondria and plastids is not possible because there are no established methods for delivering sgRNAs to these organelles. However, TALE-DdCBEs, the protein-only genome editing systems can be used for organellar genome editing. Moreover, TALEs can be placed more flexibly than Cas nucleases because they do not require the presence of a PAM sequence at a given distance to the target cytosine.

## Conclusions

In summary, we refined the targeting scope of TALE-DdCBEs and successfully applied them to target the chloroplast and nuclear genomes. The nuclear mutations in *N. benthamiana* were inherited by the next generation. These protein-only base editing tools broaden the plant genome editing toolbox and provide a valuable resource for plant organellar and nuclear DNA editing.

## Methods

### Plasmid construction

The TALE-DdCBE plasmids were generated using the modular cloning (MoClo) syntax [32, 42, 43]. All the components were subcloned into individual modules that can be assembled using Golden Gate Cloning [44]. The details of the cloning procedures are listed in Additional file 1: Fig. S5. The plasmid modules used in this study are listed in Additional file 1: Tables S3 and S4 and Supplemental Sequences.

### *Nicotiana benthamiana* infiltration and GUS reporter assay

GUS reporter assays were performed as previously described [9]. Briefly, *Agrobacterium tumefaciens* GV3101 strains containing a TALE-DdCBE construct and the GUS reporter construct were mixed 1:1 with an OD_600_ of 0.8 and inoculated into *N. benthamiana* leaves. After 2 to 3 days, two leaf discs (diameter 0.8 cm) were harvested from the inoculation spot. Leaf tissues were homogenized and incubated with 4-methyl-umbelliferyl-β-d-glucuronide. GUS activities were measured using a TECAN reader (360 nm excitation and 465 nm emission). Proteins were quantified by NanoDrop™ One (Thermo Fisher Scientific).

### Protoplast isolation and transformation

Rice cultivar Kitaake leaves were used to prepare protoplasts. Rice protoplast and *N. benthamiana* protoplast isolation and transformation were performed as previously described [45, 46]; 10 µg plasmid DNA per construct was introduced into protoplasts by PEG-mediated transfection. The transfected protoplasts were incubated at room temperature. After 48 h, the protoplasts were collected and the genomic DNA extracted.

### Plant transformation

Rice cultivar Kittake was used for *A. tumefaciens*-mediated stable transformation, as previously described [47]. Briefly, *A. tumefaciens* strains EHA105, containing left and right TALE-DdCBEs, as well as a *hygromycin* resistance gene as a selection marker, were used to transform calli. The transformed calli were transferred to plates containing 50 mg/l hygromycin for selection. Regenerated calli were then subjected to genotyping. Stable transformation of *N. benthamiana* was done as previously described [48]. Two *A. tumefaciens* GV3101 strains, one containing left TALE-DddA11-N and the *hygromycin* resistance gene as a selection marker, the other containing right TALE-DddA11-C and the *neomycin phosphotransferase II* resistance gene as a selection marker, were mixed in a 1:1 ratio prior to transformation of leaf cuts. The leaf explants were transferred to MS plates containing 25 mg/l hygromycin and 100 mg/l kanamycin for selection. Regenerated shoots were genotyped.

### DNA extraction and amplicon sequencing

We used the innuPREP Plant DNA Kit (Analytik Jena) to extract plant genomic DNA. The targeted sequences were amplified with specific primers (Additional file 1: Table S5), and the amplicons were purified with the GeneJET Gel Extraction Kit (Thermo Fisher Scientific) then quantified using a Qubit™ 1X dsDNA High Sensitivity Kit (Thermo Fisher Scientific). Oligos used in this study are listed in Additional file 1: Table S5. Equal amounts of PCR products were pooled and sequenced (GENEWIZ, AMPLICON-EZ). Amplicon deep sequencing was performed three times for each target location using genomic DNA isolated from three different protoplast transformation experiments. The target sites in the sequenced reads were analyzed for mutations using CRISPResso2 (Additional file 1: Table S6) [49].

### Off-target site prediction

Off-target sites were predicted by the online tool TALENoffer [50] with parameters set to a minimum and maximum distance of 4 and 20 between the TALEs. Based on the off-target score, the top 5 predicted off-target sites were selected as potential off-target sites (Additional file 1: Table S2). Site-specific primers were used to amplify the potential off-target sites. The PCR products were purified and Sanger sequenced.

### Plant growth condition

*Nicotiana benthamiana* plants were grown in a greenhouse with 16 h of light, a relative humidity of 40–60%, and temperatures of 23 °C and 19 °C during day and night, respectively. Four- to 6-week-old plants were used for *A. tumefaciens* inoculation experiments.

### Statistical analysis

All values are shown as means ± SEM (standard error of the mean). Statistical differences between the values were tested using two-tailed unpaired Student’s *t*-tests by GraphPad (Prism; www.graphpad.com).

### Supplementary Information

**Additional file 1.** **Fig. S1.** Schematic of the GUS^G537^ cytosine base editing reporter. **Fig. S2.** Editing efficiency of TALE-DdCBEs with fusion of Rad51. **Fig. S3.** DddA variants showed enhanced editing in the GUS^G537^ reporter. **Fig. S4.** TALE-DdCBE11- induced C•G-to-T•A editing in the rice chloroplast genome. **Fig. S5.** Schematic illustration for assembling TALE-DdCBEs. **Table S1.** Inheritance of mutations in T1 lines. **Table S2.** Analyzing potential off-target editing of TALE-DdCBE11 in T0 *N. benthamiana* plants. **Table S3.** Plasmids used in this study. **Table S4.** TALE binding sequences and corresponding RVDs. **Table S5.** Oligos used in this study. **Table S6.** Alignment of amplicon sequencing results. Supplemental Sequences. Amino acid sequences of TALE-DdCBE architectures.

## Data Availability

The amplicon sequencing data have been deposited in an NCBI BioProject database: PRJNA950930 [51].

## Abbreviations

TALE: Transcription activator-like effector
TALE-DdCBEs: TALE-derived DddA-based cytosine base editors
DddA: Double-strand DNA cytidine deaminase
GUS: β-Glucuronidase
SNVs: Single nucleotide variants
dsDNA: Double-stranded DNA
ssDNA: Single-stranded DNA
CBEs: Cytosine base editors
ABEs: Adenine base editors
UGI: Uracil glycosylase inhibitor
MoClo: Modular cloning
