# Supplementary material for: Targeted C•G-to-T•A base editing with TALE-cytosine deaminases in plants
Source: BMC Biol. 2024 Apr 29;22:99. doi: 10.1186/s12915-024-01895-0 (PMC11057107; doi:10.1186/s12915-024-01895-0)
Supplement: Supplementary file 1 — Additional file 1. Fig. S1. Schematic of the GUSG537 cytosine base editing reporter. Fig. S2. Editing efficiency of TALE-DdCBEs with fusion of Rad51. Fig. S3. DddA variants showed enhanced editing in the GUSG537 reporter. Fig. S4. TALE-DdCBE11- induced C•G-to-T•A editing in the rice chloroplast genome. Fig. S5. Schematic illustration for assembling TALE-DdCBEs. Table S1. Inheritance of mutations in T1 lines. Table S2. Analyzing potential off-target editing of TALE-DdCBE11 in T0 N. benthamiana plants. Table S3. Plasmids used in this study. Table S4. TALE binding sequences and corresponding RVDs. Table S5. Oligos used in this study. Table S6. Alignment of amplicon sequencing results. Supplemental Sequences. Amino acid sequences of TALE-DdCBE architectures. [file 12915_2024_1895_MOESM1_ESM.pdf]

Targeted C•G-to-T•A base editing with TALE cytosine deaminases in plants

Dingbo Zhang, Vanessa Pries, Jens Boch*

Leibniz University Hannover, Institute of Plant Genetics, Herrenhäuser Str. 2, 30419 Hannover, Germany

Additional file 1

Figure S1. Schematic of the GUS^{G537} cytosine base editing reporter.

Figure S2. Editing efficiency of TALE-DdCBEs with fusion of Rad51.

Figure S3. DddA variants showed enhanced editing in the GUS^{G537} reporter.

Figure S4. TALE-DdCBE11-induced C•G-to-T•A editing in the rice chloroplast genome.

Figure S5. Schematic illustration for assembling TALE-DdCBEs.

Table S1. Inheritance of mutations in T1 lines.

Table S2. Analyzing potential off-target editing of TALE-DdCBE11 in T0 *N. benthamiana* plants.

Table S3. Plasmids used in this study.

Table S4. TALE binding sequences and corresponding RVDs.

Table S5. Oligos used in this study.

Table S6. Alignment of amplicon sequencing results.

Supplemental Sequences. Amino acid sequences of TALE-DdCBE architectures.

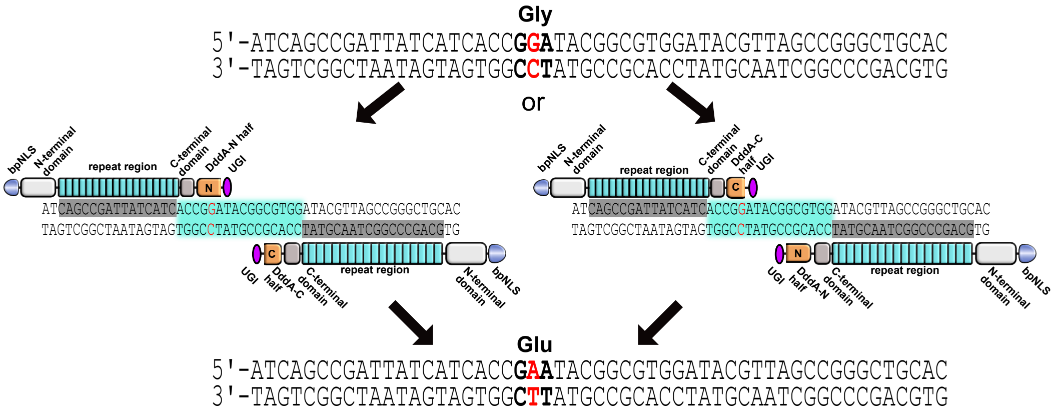

Figure S1. Schematic of the GUS^{G537} cytosine base editing reporter.
The C•G-to-T•A (highlight in red) editing in GUS^{G537} can alter the glycine (GGA) to glutamic acid (GAA) and restore GUS activity. TALE binding sites are in gray background, spacer region in cyan background, respectively.

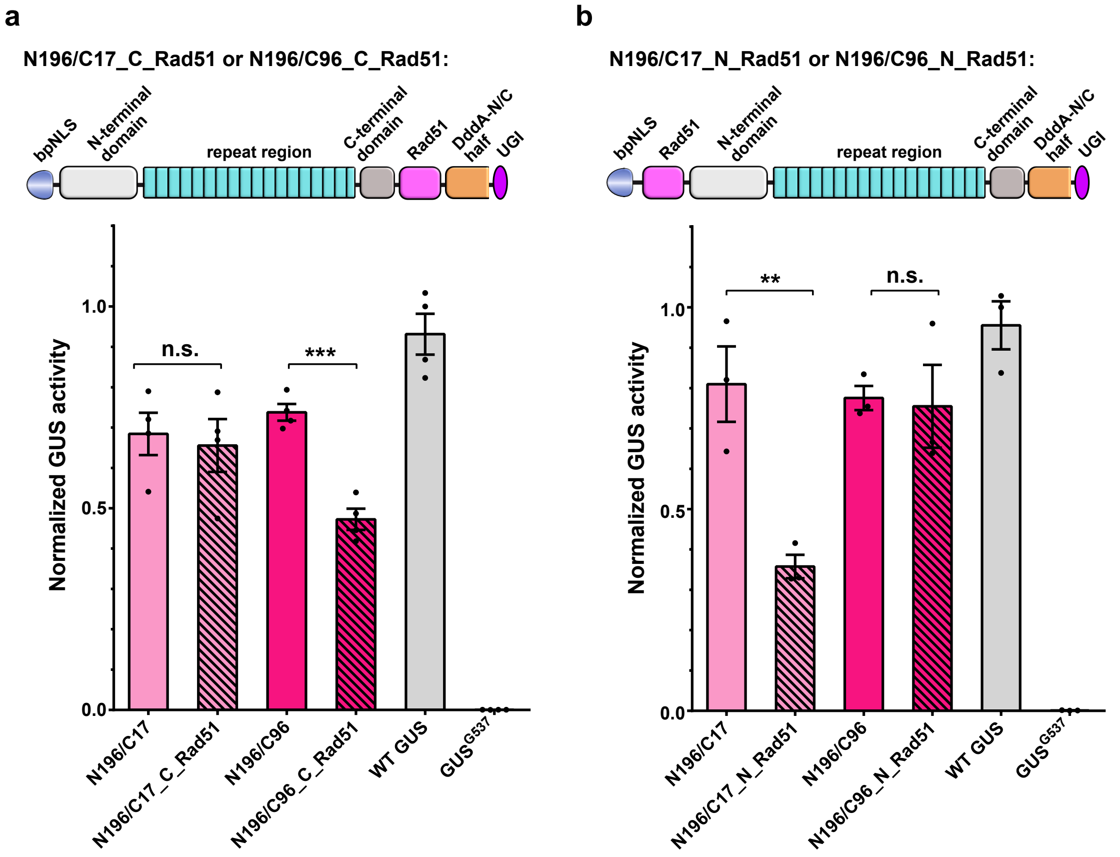

Figure S2. Editing efficiency of TALE-DdCBEs with fusion of Rad51.

(a) C•G-to-T•A editing efficiencies of TALE-DdCBEs with Rad51 inserted between TALE C-terminal domain and DddA halves in GUS^{G537} reporter, n = 4. **(b)** C•G-to-T•A editing efficiencies of TALE-DdCBEs with Rad51 inserted between bpNLS and TALE N-terminal domain in GUS^{G537} reporter, n = 3. bpNLS: bipartite nuclear localization sequence. UGI: uracil glycosylase inhibitor. GUS activities were measured and normalized to 2x35S::GUS (WT GUS, positive control). Values and error bars indicate the mean ± SEM, ***$p < 0.001$, **$p < 0.01$, n.s.: not significant, using Student's two-tailed unpaired t-test.

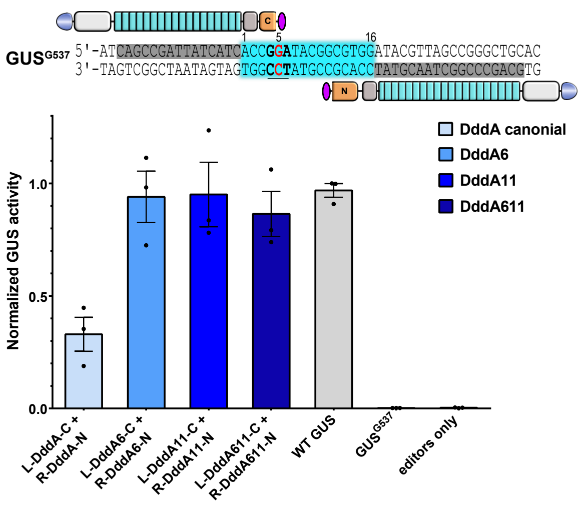

Figure S3. DddA variants showed enhanced editing in the GUS^{G537} reporter.

C•G-to-T•A editing efficiencies of TALE-DdCBEs harboring canonical DddA or DddA6 or DddA11 or DddA611 in GUS^{G537} reporter. Top: TALE-DdCBEs using left TALE-DddA-C / right TALE-DddA-N architecture, targeted cytosine located at C5 within the 16-nt spacer. Bottom: values and error bars indicate the mean ± SEM, n=3.

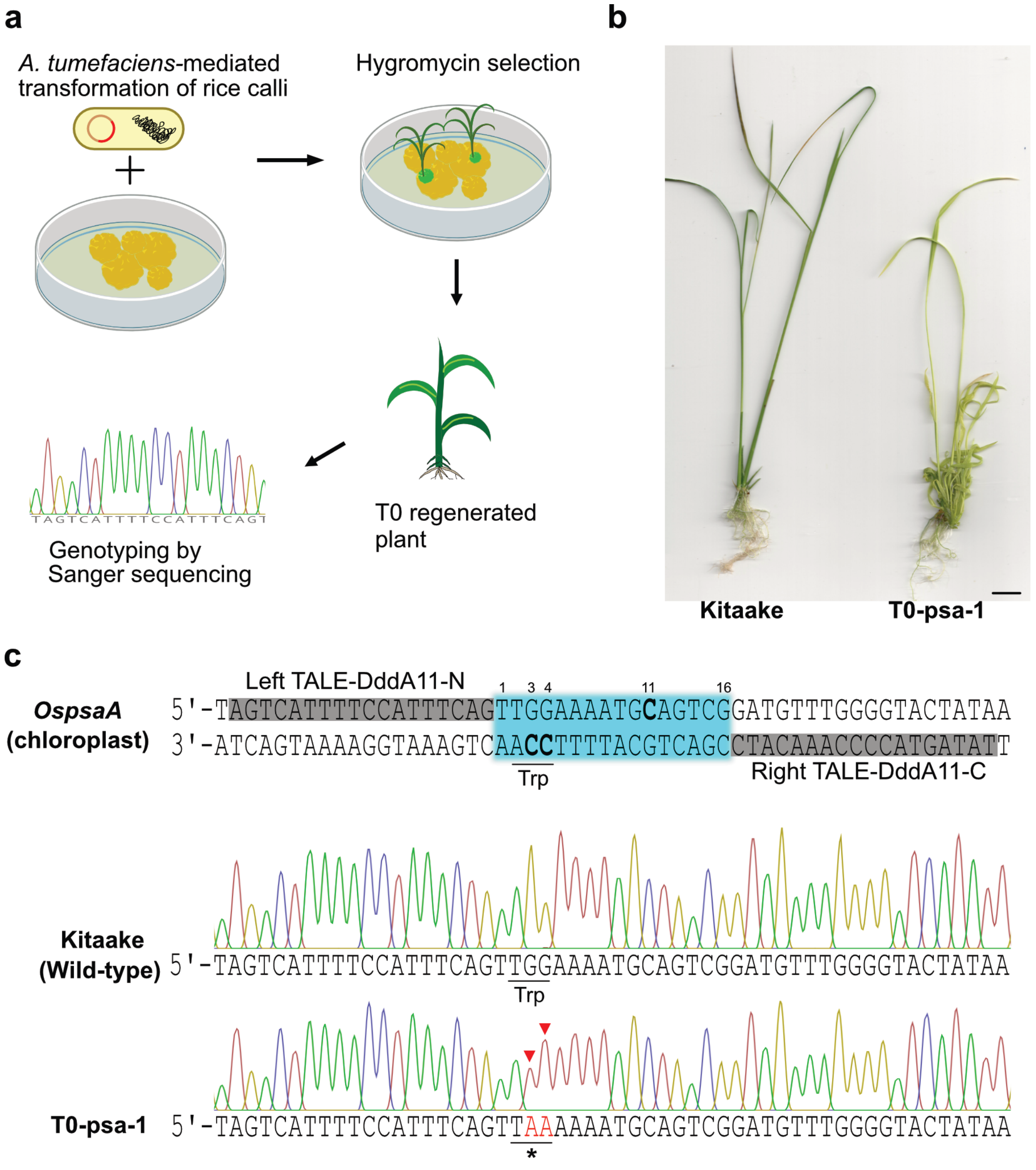

Figure S4. TALE-DdCBE11-induced C•G-to-T•A editing in the rice chloroplast genome. (a) Schematic C•G-to-T•A editing mediated by TALE-DdCBE11 in rice plants. **(b)** Phenotypes of the *OspsaA* mutant line T0-psa-1 and a wild-type Kitaake plant. Bar = 1 cm. **(c)** Genotypes of line T0-psa-1 and wild-type Kitaake plants. A pair of TALE-DddA11 was targeting the rice *OspsaA* chloroplast gene. TALE-binding sites are in gray background and the spacer region is in cyan background. Sanger sequencing chromatograms of wild-type and regenerated T0 plants are shown. Base conversions are indicated in red and marked by red triangles. The tryptophan (Trp) codon TGG was converted to a TAA stop codon (*).

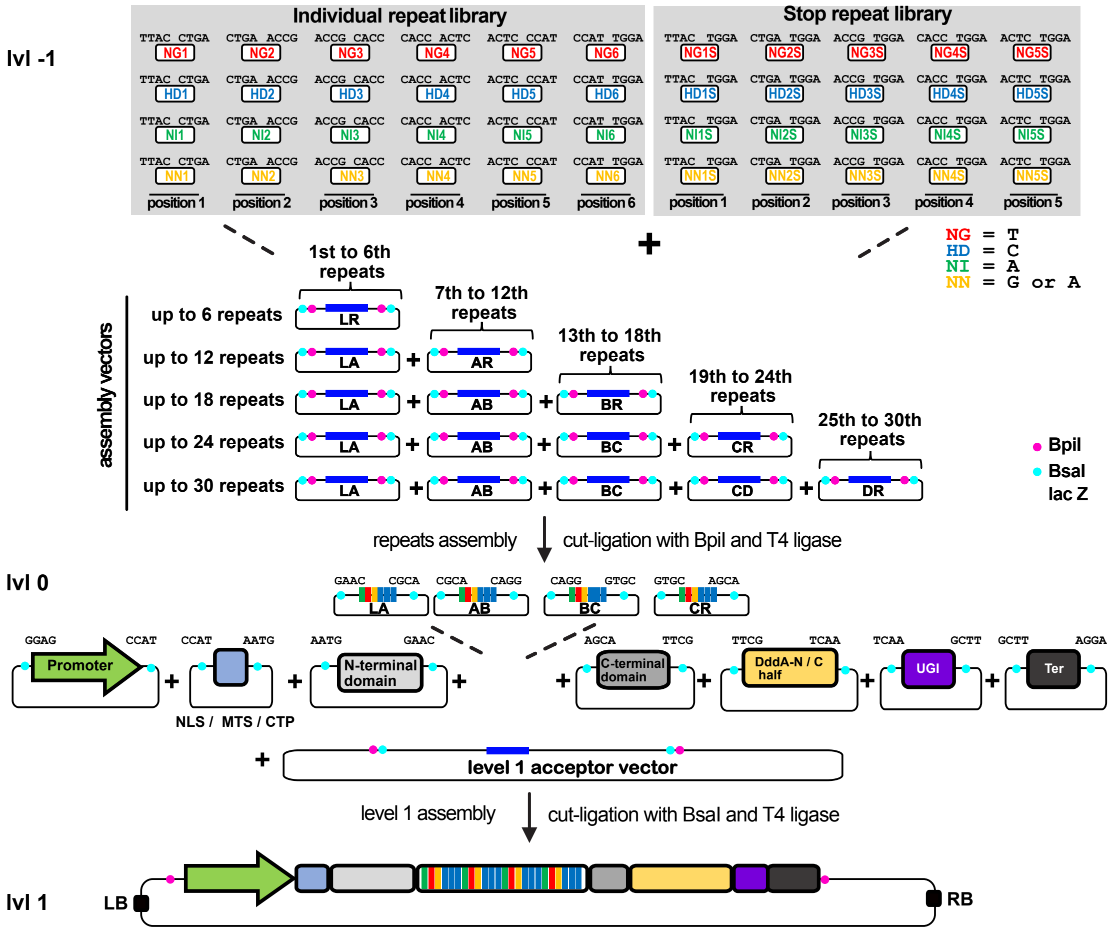

Figure S5. Schematic illustration for assembling TALE-DdCBEs.
Golden-Gate assembly of TALE-DdCBEs based on the MoClo design. Corresponding to the target sequence (with a thymine at the 5’ end), up to six single repeats are ligated into the corresponding receiving plasmids. Next, those multi-repeat modules are combined with promoter, NLS or MTS or CTP, N-terminal domain, C-terminal domain, DddA half, terminator (Ter) and level 1 acceptor vector to yield the level 1 TALE-DdCBEs transcription unit. Level 1 vectors are using for transient transformation (protoplasts assay and infiltration of *N. benthamiana* leaves). Four-base overhang sequences are shown on the modules. NLS: Nuclear localization signal. MTS: Mitochondrial targeting signal. CTP: chloroplast transition peptide.

Table S1. Inheritance of mutations in T1 *N. benthamiana* lines.

Target gene	T0 lines	T0 genotypes†	Number of T1 plants	Number, and genotypes of T1 plants†
NbSuRB	T0-6	C6->T (He)	4	4, C6->T (He)
	T0-7	C6->T (He)	2	1, C6->T (He);<br>1, C6->T (Ho)
	T0-9	C6->G (He)	4	2, C6->G (He);<br>2, C6->G (Ho)
	T0-10	C6->T (He),<br>C7->T (He)	2	1, C6->T (Ho) & C7->T (Ho)<br>1, C6->T (Ho) & C7->T (He)

†: He: Heterozygous. Ho: Homozygous.

Table S2. Analyzing potential off-target editing of TALE-DdCBE11 in T0 *N. benthamiana* plants.

Target sites	Left TALE binding sequence (5'-3')†	Spacer (5'-3')	Right TALE binding sequence (5'-3')†	Off-target editing (line No., editing)
On-target (*NbSuRB*)	TGTCATGCTGGGTC	AGATTGGAACTT	TCCAACCTAAAGAGG	-
Off-target site 1	TCTCATGCTTCTTC	CTTCAACTT	TCCAACCTAAAAAGG	T0-3, not detected
				T0-6, not detected
				T0-10, not detected
Off-target site 2	TATCATGCAGAGTT	TGATTGCA	TCCAACCTAAAGAAG	T0-3, not detected
				T0-6, not detected
				T0-10, not detected
Off-target site 3	TGTCATGCTGGGTC	AAACCTCGACCTTTCC	CACAACACAAAGACG	T0-3, not detected
				T0-6, not detected
				T0-10, not detected
Off-target site 4	TATCATGCTGGGTC	AACCTTTATGACCTTTCC	CACAACATAAAGATG	T0-3, not detected
				T0-6, not detected
				T0-10, not detected
Off-target site 5	TATCATGATGAGTT	TTCCATCA	TCCAACCTAAAAAAG	T0-3, not detected
				T0-6, not detected
				T0-10, not detected

†: Mismatched sequences are highlighted in red.

Table S3. Plasmids used in this study.

Name	MoClo level	Content
L0_35s_Pro5U	level 0	2X35S promoter with GGAG/CCAT overhang
L0_ZmUbi_Pro5U	level 0	Maize ubiquitin promoter with GGAG/CCAT overhang
L0_Noster	level 0	Nos terminator with GCTT/AGGA overhang
L0_35s_ter	level 0	35S terminator with GCTT/AGGA overhang
L0_bpNLS	level 0	bpNLS with CCAT/AATG overhang
L0_TND_288	level 0	TALE N-Terminal domain (288 aa) with AATG/GAAC overhang
L0_TND_196	level 0	TALE N-Terminal domain (196 aa) with AATG/GAAC overhang
L0_TND_135	level 0	TALE N-Terminal domain (135 aa) with AATG/GAAC overhang
L0_TCD_17	level 0	TALE C-Terminal domain (17 aa) with AGCA/TTCG overhang
L0_TCD_28	level 0	TALE C-Terminal domain (28 aa) with AGCA/TTCG overhang
L0_TCD_63	level 0	TALE C-Terminal domain (63 aa) with AGCA/TTCG overhang
L0_TCD_96	level 0	TALE C-Terminal domain (96 aa) with AGCA/TTCG overhang
L0_TCD_247	level 0	TALE C-Terminal domain (247 aa) with AGCA/TTCG overhang
L0_UGI	level 0	UGI-NLS with TCAA/GCTT overhang
L0_CTP	level 0	Chloroplast transition peptide with CCAT/AATG overhang
L0_DddA_N	level 0	DddA-N half with TTCG/TCAA overhang
L0_DddA_C	level 0	DddA-C half with TTCG/TCAA overhang
L0_DddA6_N	level 0	DddA6-N half with TTCG/TCAA overhang
L0_DddA6_C	level 0	DddA6-C half with TTCG/TCAA overhang
L0_DddA11_N	level 0	DddA11-N half with TTCG/TCAA overhang
L0_DddA11_C	level 0	DddA11-C half with TTCG/TCAA overhang
L0_DddA611_N	level 0	DddA611-N half with TTCG/TCAA overhang
L0_DddA611_C	level 0	DddA611-C half with TTCG/TCAA overhang
L0_DddA6^{K1389A}_N	level 0	DddA6^{K1389A} -N half with TTCG/TCAA overhang
L0_DddA6^{K1389A} _C	level 0	DddA6^{K1389A} -C half with TTCG/TCAA overhang
L0_DddA6^{T1391A}_N	level 0	DddA6^{T1391A} -N half with TTCG/TCAA overhang
L0_DddA6 ^{T1391A} _C	level 0	DddA6^{T1391A} -C half with TTCG/TCAA overhang
L0_DddA11^{K1389A}_N	level 0	DddA11^{K1389A} -N half with TTCG/TCAA overhang
L0_DddA11^{K1389A}_C	level 0	DddA11^{K1389A} -C half with TTCG/TCAA overhang

L0_DddA11^{T1391A}_N	level 0	DddA11^{T1391A} -N half with TTCG/TCAA overhang
L0_DddA11^{T1391A} _C	level 0	DddA11^{T1391A} -C half with TTCG/TCAA overhang
L0_DddA_N_UGI	level 0	DddA-N half-UGI-NLS fusion with TGGC/AGGA overhang
L0_DddA_C_UGI	level 0	DddA-C half-UGI-NLS fusion with TGGC/AGGA overhang
L0_NLS_Rad51_N	level 0	bpNLS-Rad51 fusion with CCAT/AATG overhang
L0_Rad51_C	level 0	Rad51 domain with TTCG/TGGC overhang
pICH47732_DZ	level 1 accepter	MoClo level 1 accepter in position 1 with GGAG/AGGA overhang
pICH47742_DZ	level 1 accepter	MoClo level 1 accepter in position 2 with GGAG/AGGA overhang
pGUS_WT	level M	Wild-type GUS expression plasmid containing a p19 silencing suppressor
pGUS_537	level M	GUS^{G537} expression plasmid containing a p19 silencing suppressor
For TALE repeats see Geiβler et al., 2011		

Table S4. TALE binding sequences and corresponding RVDs.

Gene	Binding sequence † (5'-3')	RVDs
GUS^{G537}-left-TALE1	TCAGCCGATTATC	HD NI NN HD HD NN NI NG NG NI NG HD
GUS^{G537}-left-TALE2	TCAGCCGATTATCA	HD NI NN HD HD NN NI NG NG NI NG HD NI
GUS^{G537}-left-TALE3	TCAGCCGATTATCAT	HD NI NN HD HD NN NI NG NG NI NG HD NI NG
GUS^{G537}-left-TALE4	TCAGCCGATTATCATC	HD NI NN HD HD NN NI NG NG NI NG HD NI NG HD
GUS^{G537}-left-TALE5	TCAGCCGATTATCATCA	HD NI NN HD HD NN NI NG NG NI NG HD NI NG HD NI
GUS^{G537}-left-TALE6	TCAGCCGATTATCATCAC	HD NI NN HD HD NN NI NG NG NI NG HD NI NG HD NI HD
GUS^{G537}-left-TALE7	TCAGCCGATTATCATCACC	HD NI NN HD HD NN NI NG NG NI NG HD NI NG HD NI HD HD
GUS^{G537}-left-TALE8	TCAGCCGATTATCATCACCG	HD NI NN HD HD NN NI NG NG NI NG HD NI NG HD NI HD HD NN
GUS^{G537}-right TALE1	TAACGTATCCACG	NI NI HD NN NG NI NG HD HD NI HD NN
GUS^{G537}-right TALE2	TAACGTATCCACGC	NI NI HD NN NG NI NG HD HD NI HD NN HD
GUS^{G537}-right TALE3	TAACGTATCCACGCC	NI NI HD NN NG NI NG HD HD NI HD NN HD HD
GUS^{G537}-right TALE4	TAACGTATCCACGCCG	NI NI HD NN NG NI NG HD HD NI HD NN HD HD NN
GUS^{G537}-right TALE5	TAACGTATCCACGCCGT	NI NI HD NN NG NI NG HD HD NI HD NN HD HD NN NG
GUS^{G537}-right TALE6	TAACGTATCCACGCCGTA	NI NI HD NN NG NI NG HD HD NI HD NN HD HD NN NG NI
GUS^{G537}-right TALE7	TAACGTATCCACGCCGTAT	NI NI HD NN NG NI NG HD HD NI HD NN HD HD NN NG NI NG
GUS^{G537}-right TALE8	TGCAGCCCGGCTAACGTAT	NN HD NI NN HD HD HD NN NN HD NG NI NI HD NN NG NI NG
Nb-T1-left TALE	TGCATAGCGCATTAA	NN HD NI NG NI NN HD NN HD NI NG NG NI NI
Nb-T1-right TALE	TCACGAATGAGGTTG	HD NI HD NN NI NI NG NN NI NN NN NG NG NN
OsALS-T1-left TALE	TGCTTGCATTTGGCGTGCG	NN HD NG NG NN HD NI NG NG NG NN NN HD NN NG NN HD NN

OsALS-T1-right TALE	**TGCAAAAGCCTCAATTTTCCCT**	**NN HD NI NI NI NI NN HD HD NG HD NI NI NG NG NG NG HD HD HD NG**
OsALS-T2-left TALE	**TGGGTATGGTTGTGCAATG**	**NN NN NN NG NI NG NN NN NG NG NN NG NN HD NI NI NG NN**
OsALS-T2-right TALE	**TATGCGCCCTATTTGCCTT**	**NI NG NN HD NN HD HD HD NG NI NG NG NG NN HD HD NG NG**
OsPDS-left TALE	**TTCCTGGTAAATAGGTAGT**	**NG HD HD NG NN NN NG NI NI NI NG NI NN NN NG NI NN NG**
OsPDS-right TALE	**TGCTCAAAGCCAGCAGTCA**	**NN HD NG HD NI NI NI NN HD HD NI NN HD NI NN NG HD NI**
OspsaA-left TALE	**TAGTCATTTTCCATTTCAG**	**NI NN NG HD NI NG NG NG NG HD HD NI NG NG NG HD NI NN**
OspsaA-right TALE	**TTATAGTACCCCAAACATC**	**NG HI NG NI NN NG NI HD HD HD HD NI NI NI HD NI NG HD**
NbSuRB-left TALE	**TGTCATGCTGGGTC**	**NN NG HD NI NG NN HD NG NN NN NN NG HD**
NbSuRB-right TALE	**TCCAACCTAAAGAGG**	**HD HD NI NI HD HD NG NI NI NI NN NI NN NN**
unspecific-TALE	**TCACGAATGAGGTTG**	**HD NI HD NN NI NI NG NN NI NN NN NG NG NN**

Table S5. Oligos used in this study.

Name	Sequence 5' - 3'	Application
NB1-T1-AP-F1	GATATTTATGTTTGTTTTGC	Amplify NB-T1
NB1_R	CCTAGGACTCGAGAGAAAGCTGCAAATC	
OsALS-T1-AP-F1	GTGATGATCCGTTGTCCC	Amplify OsALS-T1
OsALS-T1-AP-R1	GCAAGCTTAACATCTGCG	
OsALS-T2-AP-F1	TGCAGGAATATTGAACCC	Amplify OsALS-T2
OsALS-T2-AP-R1	TATTGATGGGGATGGTAG	
OsPDS-AP-F1	CGTATCTGTATTCCGTAG	Amplify *OsPDS*
OsPDS-AP-R1	ACCTCGTTTGCCTCCTC	
PsA-F	GCTCCTGGTGCAACAACAAG	Amplify *OspsaA*
PsA-R	AGGCCCAGACAAAATGAGCA	
PsA-AP-F	TCTTGGTTTTCGCTTCCCTT	Amplify *OspsaA* for NGS
PsA-AP-R	AATTACTTGGGATGCCTGTG	
NbSURB-F	GCAGAGCTAGTTCTAGGCGTC	Amplify *NbSURB*
NbSURB-R	GGTTCTGGTTTATTGATAGAGCTGC	
NB-OT1-F	TTTTCAGCCGTGGAGGTCTC	Amplify *NbSURB* predicted off-target site 1
NB-OT1-R	TTGCCAGTGACGATCGAACA	
NB-OT2-F	ATTGAGGCCACCACCATGTT	Amplify *NbSURB* predicted off-target site 2
NB-OT2-R	TTCCCTCAAGCTCCGTCAAC	
NB-OT3-F	GGGAAACGCACCCATAGTCA	Amplify *NbSURB* predicted off-target site 3
NB-OT3-R	AAGAGTGAGGGCAATGGACG	
NB-OT4-F	ATGTCGGGGCAAGAAAGGAG	Amplify *NbSURB* predicted off-target site 4
NB-OT4-R	TGCTCGCCCATTAGTGTGTT	
NB-OT5-F	GAGGCTGCCACCATGTTTTG	Amplify *NbSURB* predicted off-target site 5
NB-OT5-R	GGTCGCACATGTCAAGCATC	

Table S6. Alignment of amplicon sequencing results.

Aligned result of amplicon sequencing results			
	**Aligned sequences**		
OsPDS reference	**CCTGGTAAATAGGTAGTAGGAGAGATGCTGAGATGACTGCTGGCTTTGAGC**ATGCGGCAT	**Reads**	**Reads (%)**
OsPDS (DddA) Repetition 1	CCTGGTAAATAGGTAGTAGGAGAGATGCTGAGATGACTGCTGGCTTTGAGCATGCGGCAT	4825	100
OsPDS (DddA) Repetition 2	CCTGGTAAATAGGTAGTAGGAGAGATGCTGAGATGACTGCTGGCTTTGAGCATGCGGCAT	14709	99.117
OsPDS (DddA) Repetition 3	CCTGGTAAATAGGTAGTAGGAGAGATGCTGAGATGACTGCTGGCTTTGAGCATGCGGCAT	14356	99.363
OsPDS (DddA11) Repetition 1	CCTGGTAAATAGGTAGTAGGAGAGATGCTGAGATGACTGCTGGCTTTGAGCATGCGGCAT	3420	95.265
	CCTGGTAAATAGGTAGTAGGAGAGATGTTGAGATGACTGCTGGCTTTGAGCATGCGGCAT	57	1.588
	CCTGGTAAATAGGTAGTAGGAAAAATGCTGAGATGACTGCTGGCTTTGAGCATGCGGCAT	7	0.195
	CCTGGTAAATAGGTAGTAGAAAAAATGCTGAGATGACTGCTGGCTTTGAGCATGCGGCAT	6	0.167
	CCTGGTAAATAGGTAGTAGGAGAGATGCTGAAATGACTGCTGGCTTTGAGCATGCGGCAT	4	0.111
	CCTGGTAAATAGGTAGTAGGAGAAATGCTGAGATGACTGCTGGCTTTGAGCATGCGGCAT	3	0.084
OsPDS (DddA11) Repetition 2	CCTGGTAAATAGGTAGTAGGAGAGATGCTGAGATGACTGCTGGCTTTGAGCATGCGGCAT	10636	97.221
	CCTGGTAAATAGGTAGTAGGAGAGATGTTGAGATGACTGCTGGCTTTGAGCATGCGGCAT	49	0.448
	CCTGGTAAATAGGTAGTAGGAAAAATGCTGAGATGACTGCTGGCTTTGAGCATGCGGCAT	8	0.073
	CCTGGTAAATAGGTAGTAGGAGAAATGCTGAGATGACTGCTGGCTTTGAGCATGCGGCAT	4	0.037
	CCTGGTAAATAGGTAGTAGAAAAAATGTTGAGATGACTGCTGGCTTTGAGCATGCGGCAT	1	0.009
	CCTGGTAAATAGGTAGTAGGAGAGATGCTGAAATGACTGCTGGCTTTGAGCATGCGGCAT	1	0.009
OsPDS (DddA11) Repetition 3	CCTGGTAAATAGGTAGTAGGAGAGATGCTGAGATGACTGCTGGCTTTGAGCATGCGGCAT	12194	95.895
	CCTGGTAAATAGGTAGTAGGAGAGATGTTGAGATGACTGCTGGCTTTGAGCATGCGGCAT	170	1.337
	CCTGGTAAATAGGTAGTAGGAAAAATGCTGAGATGACTGCTGGCTTTGAGCATGCGGCAT	34	0.267
	CCTGGTAAATAGGTAGTAGAAAAAATGCTGAGATGACTGCTGGCTTTGAGCATGCGGCAT	13	0.102
	CCTGGTAAATAGGTAGTAGGAGAAATGCTGAGATGACTGCTGGCTTTGAGCATGCGGCAT	10	0.079

	CCTGGTAAATAGGTAGTAGGAGAGATGCTGAAATGACTGCTGGCTTTGAGCATGCGGCAT	1	0.008
OsALS-T1 reference	TGTT**GCTTGCATTTGGCGTGCGGTTTGATGATCGTGTGACAGGGAAAATTGAGGCTTTTG**	**Reads**	**Reads (%)**
OsALS-T1 (DddA) Repetition 1	TGTTGCTTGCATTTGGCGTGCGGTTTGATGATCGTGTGACAGGGAAAATTGAGGCTTTTG	5043	93.216
	TGTTGCTTGCATTTGGCGTGCGGTTTGATGATTGTGTGACAGGGAAAATTGAGGCTTTTG	108	1.996
OsALS-T1 (DddA) Repetition 2	TGTTGCTTGCATTTGGCGTGCGGTTTGATGATCGTGTGACAGGGAAAATTGAGGCTTTTG	5174	93.141
	TGTTGCTTGCATTTGGCGTGCGGTTTGATGATTGTGTGACAGGGAAAATTGAGGCTTTTG	158	2.844
OsALS-T1 (DddA) Repetition 3	TGTTGCTTGCATTTGGCGTGCGGTTTGATGATCGTGTGACAGGGAAAATTGAGGCTTTTG	7477	92.663
	TGTTGCTTGCATTTGGCGTGCGGTTTGATGATTGTGTGACAGGGAAAATTGAGGCTTTTG	275	3.408
OsALS-T1 (DddA11) Repetition 1	TGTTGCTTGCATTTGGCGTGCGGTTTGATGATCGTGTGACAGGGAAAATTGAGGCTTTTG	4067	95.291
	TGTTGCTTGCATTTGGCGTGCGGTTTGATGATTGTGTGACAGGGAAAATTGAGGCTTTTG	124	2.905
OsALS-T1 (DddA11) Repetition 2	TGTTGCTTGCATTTGGCGTGCGGTTTGATGATCGTGTGACAGGGAAAATTGAGGCTTTTG	14381	95.937
	TGTTGCTTGCATTTGGCGTGCGGTTTGATGATTGTGTGACAGGGAAAATTGAGGCTTTTG	352	2.348
OsALS-T1 (DddA11) Repetition 3	TGTTGCTTGCATTTGGCGTGCGGTTTGATGATCGTGTGACAGGGAAAATTGAGGCTTTTG	15817	94.741
	TGTTGCTTGCATTTGGCGTGCGGTTTGATGATTGTGTGACAGGGAAAATTGAGGCTTTTG	569	3.408
	TGTTGCTTGCATTTGGCGTGCGGTTTGATGATAGTGTGACAGGGAAAATTGAGGCTTTTG	24	0.144
OsALS-T2 reference	**GGTATGGTTGTGCAATGGGAGGATAGGTTTTACAAGGCAAATAGGGCGCAT**ACATACTTG	**Reads**	**Reads (%)**
OsALS-T2 (DddA)	GGTATGGTTGTGCAATGGGAGGATAGGTTTTACAAGGCAAATAGGGCGCATACATACTTG	4079	99.585
OsALS-T2 (DddA)	GGTATGGTTGTGCAATGGGAGGATAGGTTTTACAAGGCAAATAGGGCGCATACATACTTG	6819	99.810
OsALS-T2 (DddA) Repetition 3	GGTATGGTTGTGCAATGGGAGGATAGGTTTTACAAGGCAAATAGGGCGCATACATACTTG	7443	99.612
	GGTATGGTTGTGCAATGGGAGGATAAGTTTTACAAGGCAAATAGGGCGCATACATACTTG	2	0.0003
OsALS-T2 (DddA11) Repetition 1	GGTATGGTTGTGCAATGGGAGGATAGGTTTTACAAGGCAAATAGGGCGCATACATACTTG	5252	93.336
	GGTATGGTTGTGCAATGGGAGGATAAATTTTACAAGGCAAATAGGGCGCATACATACTTG	233	4.141
	GGTATGGTTGTGCAATGGGAGAATAGGTTTTACAAGGCAAATAGGGCGCATACATACTTG	13	0.231
	GGTATGGTTGTGCAATGGGAGGATAAGTTTTACAAGGCAAATAGGGCGCATACATACTTG	9	0.160

OsALS-T2 (DddA11) Repetition 2	GGTATGGTTGTGCAATGGGAGGATAGGTTTTACAAGGCAAATAGGGCGCATACATACTTG	18009	97.7104
	GGTATGGTTGTGCAATGGGAGGATAAATTTTACAAGGCAAATAGGGCGCATACATACTTG	116	0.629
	GGTATGGTTGTGCAATGGGAGAATAGGTTTTACAAGGCAAATAGGGCGCATACATACTTG	3	0.0163
	GGTATGGTTGTGCAATGGGAGGATATGTTTTACAAGGCAAATAGGGCGCATACATACTTG	2	0.011
OsALS-T2 (DddA11) Repetition 3	GGTATGGTTGTGCAATGGGAGGATAGGTTTTACAAGGCAAATAGGGCGCATACATACTTG	20117	95.143
	GGTATGGTTGTGCAATGGGAGGATAAATTTTACAAGGCAAATAGGGCGCATACATACTTG	602	2.847
	GGTATGGTTGTGCAATGGGAGAATAGGTTTTACAAGGCAAATAGGGCGCATACATACTTG	22	0.104
	GGTATGGTTGTGCAATGGGAGTATAGGTTTTACAAGGCAAATAGGGCGCATACATACTTG	7	0.0331
	GGTATGGTTGTGCAATGGGAGGATAGTTTTTACAAGGCAAATAGGGCGCATACATACTTG	2	0.009
	GGTATGGTTGTGCAATGGGAGGATAGATTTTACAAGGCAAATAGGGCGCATACATACTTG	2	0.009
	GGTATGGTTGTGCAATGGGAGGATAAATTTTACAAGGCAAATAGGTCGCATACATACTTG	2	0.009
NB-T1 reference	AGT**TGCATAGCGCATTAA**TTGCGTGATCTG**CAACCTCATTCGTGA**AAAAGATCTTCTCCA	**Reads**	**Reads (%)**
NB-T1 (DddA) Repetition 1	AGTTGCATAGCGCATTAATTGCGTGATCTGCAACCTCATTCGTGAAAAAGATCTTCTCCA	16230	95.297
	AGTTGCATAGCGCATTAATTGCGTGATTTGCAACCTCATTCGTGAAAAAGATCTTCTCCA	386	2.266
	AGTTGCATAGCGCATTAATTGCGTGATATGCAACCTCATTCGTGAAAAAGATCTTCTCCA	16	0.094
	AGTTGCATAGCGCATTAATTGCGTAATCTGCAACCTCATTCGTGAAAAAGATCTTCTCCA	11	0.065
NB-T1 (DddA) Repetition 2	AGTTGCATAGCGCATTAATTGCGTGATCTGCAACCTCATTCGTGAAAAAGATCTTCTCCA	8557	95.036
	AGTTGCATAGCGCATTAATTGCGTGATTTGCAACCTCATTCGTGAAAAAGATCTTCTCCA	228	2.532
	AGTTGCATAGCGCATTAATTGCGTAATCTGCAACCTCATTCGTGAAAAAGATCTTCTCCA	1	0.011
NB-T1 (DddA) Repetition 3	AGTTGCATAGCGCATTAATTGCGTGATCTGCAACCTCATTCGTGAAAAAGATCTTCTCCA	13801	94.748
	AGTTGCATAGCGCATTAATTGCGTGATTTGCAACCTCATTCGTGAAAAAGATCTTCTCCA	355	2.437
NB-T1 (DddA6) Repetition 1	AGTTGCATAGCGCATTAATTGCGTGATCTGCAACCTCATTCGTGAAAAAGATCTTCTCCA	13071	95.256
	AGTTGCATAGCGCATTAATTGCGTGATTTGCAACCTCATTCGTGAAAAAGATCTTCTCCA	355	2.587
	AGTTGCATAGCGCATTAATTGCGTAATCTGCAACCTCATTCGTGAAAAAGATCTTCTCCA	9	0.066

NB-T1 (DddA6) Repetition 2	AGTTGCATAGCGCATTAATTGCGTGATCTGCAACCTCATTCGTGAAAAAGATCTTCTCCA	14799	95.207
	AGTTGCATAGCGCATTAATTGCGTGATTTGCAACCTCATTCGTGAAAAAGATCTTCTCCA	496	3.191
	AGTTGCATAGCGCATTAATTGCGTAATCTGCAACCTCATTCGTGAAAAAGATCTTCTCCA	4	0.026
NB-T1 (DddA6) Repetition 3	AGTTGCATAGCGCATTAATTGCGTGATCTGCAACCTCATTCGTGAAAAAGATCTTCTCCA	8837	95.032
	AGTTGCATAGCGCATTAATTGCGTGATTTGCAACCTCATTCGTGAAAAAGATCTTCTCCA	322	3.463
	AGTTGCATAGCGCATTAATTGCGTAATCTGCAACCTCATTCGTGAAAAAGATCTTCTCCA	3	0.032

Supplemental Sequences. Amino acid sequences of TALE-DdCBE architectures.

TALE-DdCBE (from N- to C-terminus):

bpNLS-N-terminal domain-**TALE repeats**-C-terminal domain-GS linker-DddA-N/C half-SGGS linker-UGI-NLS

bpNLS:
MKRTADGSEFESPKKKRKV

N-terminal domain
N288:
MDPIRSRTPSPARELLSGPQPDGVQPTADRGVSPPAGGPLDGLPARRTMSRTRLPSPPAPSPAFS
ADSFSDLLRQFDPSLFNTSLFDSLPPFGAHHTEAATGEWDEVQSGLRAADAPPPTMRVAVTAARP
PRAKPAPRRRAAQPSDASPAAQVDLRTLGYSQQQQEKIKPKVRSTVAQHHEALVGHGFTHAHIVA
LSQHPAALGTVAVKYQDMIAALPEATHEAIVGVGKQWSGARALEALLTVAGELRGPPLQLDTGQL
LKIAKRGGVTAVEAVHAWRNALTGAPLN
N196:
MAHHTEAATGEWDEVQSGLRAADAPPPTMRVAVTAARPPRAKPAPRRRAAQPSDASPAAQVDLRT
LGYSQQQQEKIKPKVRSTVAQHHEALVGHGFTHAHIVALSQHPAALGTVAVKYQDMIAALPEATH
EAIVGVGKQWSGARALEALLTVAGELRGPPLQLDTGQLLKIAKRGGVTAVEAVHAWRNALTGAPL
N
N135:
DLRTLGYSQQQQEKIKPKVRSTVAQHHEALVGHGFTHAHIVALSQHPAALGTVAVKYQDMIAALP
EATHEAIVGVGKQWSGARALEALLTVAGELRGPPLQLDTGQLLKIAKRGGVTAVEAVHAWRNALT
GAPLN

C-terminal domain
C17:
SIVAQLSRPDPSLAALT
C28:
SIVAQLSRPDPSLAALTNDHLVALACLG
C47:
SIVAQLSRPDPSLAALTNDHLVALACLGGRPALDAVKKGLPHAPALI
C63:
SIVAQLSRPDPSLAALTNDHLVALACLGGRPALDAVKKGLPHAPALIKRTNRRIPERTSHRVA
C96:
SIVAQLSRPDPALAALTNDHLVALACLGGRPALDAVKKGLPHAPALIKRTNRRIPERTSHRVADH
AQVVRVLGFFQCHSHPAQAFDDAMTQFGMS
C247:
SIVAQLSRPDPALAALTNDHLVALACLGGRPALDAVKKGLPHAPALIKRTNRRIPERTSHRVADH
AQVVRVLGFFQCHSHPAQAFDDAMTQFGMSRHGLLQLFRRVGVTELEARSGTLPPASQRWDRILQ
ASGMKRAKPSPTSTQTPDQASLHAFADSLERDLDAPSPMHEGDQTRASSRKRSRSDRAVTGPSAQ
QSFEVRVPEQRDALHLPLLSWGVKRPRTRIGGLLDPGTPMDADLVASSTVVW

DddA-N:

GSYALGPYQISAPQLPAYNGQTVGTFYYVNDAGGLESKVFSSGGPTPYPNYANAGHVEGQSALFM
RDNGISEGLVFHNNPEGTCGFCVNMTETLLPENAKMTVVPPEG

DddA-C:

AIPVKRGATGETKVFTGNSNSPKSPTKGGCSGGS

DddA6-N:

GSYALGPYQISAPQLPAYNGRTVGTFYYVNDAGGLESKVFISGGPTPYPNYANAGHVEGQSALFM
RDNGISEGLVFHNNPEGTCGFCVNMIETLLPENAKMTVVPPEG

DddA6-C / DddA6^{K1389A}-C / DddA6^{T1391A}-C:

AIPVKRGATGETKVFIGNSNSPKSPTKGGCSGGS

DddA6^{K1389A}-N:

GSYALGPYQISAPQLPAYNGRTVGTFYYVNDAGGLESKVFISGGPTPYPNYANAGHVEGQSALFM
RDNGISEGLVFHNNPEGTCGFCVNMIETLLPENAAMTVVPPEG

DddA6^{T1391A}-N:

GSYALGPYQISAPQLPAYNGRTVGTFYYVNDAGGLESKVFISGGPTPYPNYANAGHVEGQSALFM
RDNGISEGLVFHNNPEGTCGFCVNMIETLLPENAKMAVVPPEG

DddA11-N:

GSYALGPYQISAPQLPAYNGQTVGTFYYVNDAGGLESKVFISGGPTPYPNYVSAGHVEGQSALFM
RDNGISEGLVFHNNPKGTCGFCVNMIETLLPENAKMTVVPPEG

DddA11-C / DddA11^{K1389A}-C / DddA11^{T1391A}-C:

AIPVKRGATGETKVFIGNSNSPKSPTKGGCSGGS

DddA11^{K1389A}-N:

GSYALGPYQISAPQLPAYNGQTVGTFYYVNDAGGLESKVFISGGPTPYPNYVSAGHVEGQSALFM
RDNGISEGLVFHNNPKGTCGFCVNMIETLLPENAAMTVVPPEG

DddA11^{T1391A}-N:

GSYALGPYQISAPQLPAYNGQTVGTFYYVNDAGGLESKVFISGGPTPYPNYVSAGHVEGQSALFM
RDNGISEGLVFHNNPKGTCGFCVNMIETLLPENAKMAVVPPEG

UGI-NLS:

TNLSDIIEKETGKQLVIQESILMLPEEVEEVIGNKPESDILVHTAYDESTDENVMLLTSDAPEYK
PWALVIQDSNGENKIKMLSGGSPKKKRKV

Chloroplast transition peptide:

MAPSVMASSATTVAPFQGLKSTAGMPVARRSGNSSFGNVSNGGRIRCMQVWPIEGIKKFETLSYL
PPLGNSSFGNVSNGGRIRC
